# Soluble TNF-Alpha-Receptors I Are Prognostic Markers in TIPS-Treated Patients with Cirrhosis and Portal Hypertension

**DOI:** 10.1371/journal.pone.0083341

**Published:** 2013-12-26

**Authors:** Jonel Trebicka, Aleksander Krag, Stefan Gansweid, Peter Schiedermaier, Holger M. Strunk, Rolf Fimmers, Christian P. Strassburg, Fleming Bendtsen, Søren Møller, Tilman Sauerbruch, Ulrich Spengler

**Affiliations:** 1 Department of Internal Medicine I, University of Bonn, Bonn, Germany; 2 Gastrounit, Medical Division, Hvidovre Hospital, University of Copenhagen, Copenhagen, Denmark; 3 Department of Gastroenterology, Odense University Hospital, University of Southern Denmark, Odense, Denmark; 4 Department of Radiology, University of Bonn, Bonn, Germany; 5 Institute for Medical Biometry, Informatics and Epidemiology, University of Bonn, Bonn, Germany; 6 Centre of Functional Imaging and Research, Department of Clinical Physiology and Nuclear Medicine, Hvidovre Hospital, University of Copenhagen, Copenhagen, Denmark; University of Sydney, Australia

## Abstract

**Background:**

TNFα levels are increased in liver cirrhosis even in the absence of infection, most likely owing to a continuous endotoxin influx into the portal blood. Soluble TNFα receptors (sTNFR type I and II) reflect release of the short-lived TNFα, because they are cleaved from the cells after binding of TNFα. The aims were to investigate the circulating levels of soluble TNFR-I and -II in cirrhotic patients receiving TIPS.

**Methods:**

Forty-nine patients with liver cirrhosis and portal hypertension (12 viral, 37 alcoholic) received TIPS for prevention of re-bleeding (n = 14), therapy-refractory ascites (n = 20), or both (n = 15). Portal and hepatic venous blood was drawn in these patients during the TIPS procedure and during the control catheterization two weeks later. sTNFR-I and sTNFR-II were measured by ELISA, correlated to clinical and biochemical characteristics.

**Results:**

Before TIPS insertion, sTNFR-II levels were lower in portal venous blood than in the hepatic venous blood, as well as in portal venous blood after TIPS insertion. No significant differences were measured in sTNFR-I levels. Hepatic venous levels of sTNFR-I above 4.5 ng/mL (p = 0.036) and sTNFR-II above 7 ng/mL (p = 0.05) after TIPS insertion were associated with decreased survival. A multivariate Cox-regression survival analysis identified the hepatic venous levels of sTNFR-I (p = 0.004) two weeks after TIPS, and Child score (p = 0.002) as independent predictors of mortality, while MELD-score was not.

**Conclusion:**

Hepatic venous levels of sTNFR-I after TIPS insertion may predict mortality in patients with severe portal hypertension.

## Introduction

Portal hypertension is a major cause of mortality and morbidity in cirrhosis regardless of its aetiology [1], [2]. Portal hypertension may lead to an impaired intestinal mucosal barrier function resulting in bacterial translocation, increased influx of endotoxin, activation of cytokines and increased shedding of their receptors in and outside the liver [3]. Furthermore, portal-venous shunting may lead to a spill over of bacterial products and cytokines, e.g. TNFα, into the systemic circulation [3], [4]. TNFα increases the intrahepatic resistance due to activation of macrophages and hepatic stellate cells, whereas it leads to extra-hepatic vasodilation and increased portal-venous inflow through induction of nitric oxide. Taken together, both processes aggravate portal hypertension [5]–[10].

TNFα has two receptors, which upon binding of TNFα dissociate from the membrane of the target cells. The shedded receptors can be detected in serum as soluble TNFα receptor I (sTNFR-I, p55, CD120A) and soluble TNFα receptor II (sTNFR2-II, p75, CD120B), and have a longer half-life than TNFα itself [5]–[8], [11]. The soluble TNFα receptors have been implicated in a variety of different liver diseases, while TNFR-I mediates apoptosis and fibrosis, TNFR-II elicits immune-modulatory effects [7], [12]–[17]. The peripheral venous levels of these receptors reflect severity of hepatic inflammation in chronic hepatitis C [18]–[23], alcoholic liver injury [24]–[28], and in metabolic disorders [22], [29], [30]. In patients hepatic venous levels of soluble TNFα receptors correlated with the portal endotoxin influx [31]. Furthermore, the levels of sTNFR-II were found to be good predictors of mortality in patients with liver cirrhosis [32]. However, the prognostic potential of sTNFRs has not yet been studied in patients with respect to severity of portal hypertension before and after TIPS-insertion. Complications of portal hypertension, such as variceal bleeding or refractory ascites, can be controlled by insertion of a transjugular intrahepatic porto-systemic shunt (TIPS) [1], [2], [33]. This procedure allows to simultaneously analyse blood from the portal and hepatic vein, reflecting processes in the intestinal compartment and the liver, respectively.

Here we investigated, whether the levels of soluble TNFRs in patients with severe portal hypertension are changed after TIPS insertion and whether they predict mortality in these patients.

## Patients and Methods

### Patients and data collection

Forty-nine patients with liver cirrhosis and severe portal hypertension referred for TIPS insertions were enrolled into the study between May 1994 and March 1999. Twelve of these patients were also included into another study, where we analyzed the endotoxin levels [31]. General clinical characteristics are displayed in Table 1. Inclusion criteria were: age between 18 and 80 years; confirmed liver cirrhosis; absence of infection; suitability for TIPS placement (secondary prophylaxis for recurrent bleeding in n = 14, therapy-refractory ascites in n = 20 and both in n = 15). The exclusion criteria were hepatic encephalopathy greater than grade I; bilirubin >5 mg/dL; variceal bleeding within the last three months prior to collection of blood samples; pulmonary arterial hypertension (>35 mmHg).

**Table 1:** Clinical parameters of the patients (n = 49) at the time of TIPS placement.

Parameters	Value
**Gender (female/male)**	16/33
Age (in years)	58 (29–80)
Child score	8 (5–12)
Child category (A/B/C)	10/25/14
MELD score	7.9 (6–18.5)
Aetiology (Alcoholic liver disease/chronic hepatitis)	37/12
Ascites (Absent/mild/severe)	14/6/29
Hepato-renal syndrome (Absent/Type 1/Type 2)	30/14/5
Patients receiving diuretics (No/yes)	16/33
Patients receiving antibiotics* (No/yes)	36/13
Oesophageal varices (Absent/grade I-II/grade III-IV)	5/33/11
Recurrent variceal bleeding (Absent/present)	20/29
Patients receiving beta-blockers (No/yes)	12/37

MELD; Model for End Stage Liver Disease, Median (range). *Antibiotics used were quinolones and penicillin for various causes (pneumonia, urinary infection, SBP-prophylaxis), and patients showed no signs of infection at TIPS placement.

### Ethics statement

The patients signed a written inform consent for the procedures in the study. The local ethical committee of the University of Bonn approved the study (029/13).

### Study design

TIPS (8–10 mm Wallstent, Boston Scientific, MA, USA) insertion was performed as previously described [34], [35]. After a mean of fourteen days, an invasive procedure was performed to check TIPS patency and its effects on portal hemodynamics [34], [35]. This procedure was routinely used in many of our TIPS patients to detect early dysfunction of bare metal stents, but was widely abandoned after 2000. Portal and hepatic venous pressures were measured invasively using a pressure transducer system (Combitrans, Braun Melsung, Germany) and a multichannel monitor (Sirecust, Siemens, Germany). The difference between these pressures was defined as the portal hepatic venous pressure gradient (PHPG). Arterial pressure and heart rate were monitored non-invasively. Biochemical parameters, as well as portal and systemic haemodynamics, were measured and recorded at TIPS placement and during the TIPS check (Table 2). Biochemical parameters were analysed using standard methods.

**Table 2:** Biochemical and haemodynamic parameters before TIPS insertion and at early invasive TIPS check after a median of fourteen days (n = 49).

Biochemical parameters (units)	Before TIPS	14 days after TIPS	p-value
**Bilirubin (mg/dL)**	1.3 (0.3–6.4)	1.7 (0.6–10.4)	0.002
CHE (U/L)	1580 (364–4480)	1730 (406–4720)	0.378
Albumin (g/L)	32 (22–53)	30 (20–49)	0.175
ALT (U/L)	15 (4–416)	21 (4–276)	0.039
γ-GT (U/L)	38 (11–407)	56 (10–348)	0.009
INR	1.28 (1.00–3.04)	1.36 (1.0–2.95)	0.003
Serum creatinine (mg/dL)	1.0 (0.6–5.4)	0.9 (0.6–3.8)	0.002
Sodium (mmol/L)	134 (121–145)	135 (124–144)	0.144
PHPG overall (mmHg)	22 (13–33)	11 (7–16) ^a^	0.0001
Systolic arterial pressure (mmHg)	114 (73–149)	115 (91–174)	0.559

Data are shown as median and range and were compared by the Wilcoxon test.

CHE, choline esterase; ALT, alanine aminotransferase; γ-GT, γ-glutamyltransferase; INR, international normalized ratio; PHPG, portal hepatic pressure gradient

### Measurement of soluble TNFα receptor levels

During the TIPS procedure, blood from the portal and hepatic veins was collected from all patients as soon as the right branch of the portal vein was cannulated to determine levels of sTNFR-I and II. During the invasive procedure to check TIPS patency, the catheter was sequentially placed into the portal vein, then the hepatic vein in order to collect blood from portal and hepatic veins. Blood samples were centrifuged at 3000 rpm for 15 minutes at 4°C and stored at −80°C. In the plasma samples, levels of soluble tumor necrosis factor αreceptors Type I (55 kD or CD120a) and Type II (75 kD or CD120b) were measured by enzyme-amplified immunoassays following the instructions of the manufacturer (Medgenix Diagnostics, Fleurus, Belgium) [31], [32].

### Statistical analysis

Data are presented as mean ± standard deviations (SD) or medians and ranges. The Wilcoxon test was used for comparison of paired data and the Mann-Whitney test for unpaired comparisons. Correlations were analysed with the Spearman correlation coefficient. Kaplan-Meier curves were used to analyse the survival rates of patients using the Log-rank test, while transplanted patients were censored at time of transplantation. Univariate time-to-event analysis was performed to identify parameters which significantly predict survival. Cox-regression analysis (forward step-wise likelihood-quotient) using the significant predictors in the univariate analysis was performed to identify independent predictors of survival. Statistical analyses were performed using SPSS 18.0 for Windows (SPSS Inc. Chicago, IL, USA).

## Results

### Clinical and biochemical characteristics of the patients

The major clinical and demographic data are summarized in Table 1. The median age was 58 years, ranging from 29 to 80 years, and 16 of the patients were women. At admission, patients were classified as Child-Turcotte-Pugh (CTP) stage B with a median of 8 points. Their MELD scores ranged from 4.4 to 18.5 points with a median of 7.9 points. The aetiology of liver cirrhosis was alcohol abuse in 37 patients and chronic hepatitis C infection in 12 patients.

The patients received TIPS due to recurrent variceal bleeding in 14 cases, therapy of refractory ascites in 20 cases and for both indication in 15 cases. Twenty-nine patients had episodes of variceal bleeding prior to TIPS insertion. Oesophageal varices were absent in only 5 patients. Hepatorenal syndrome (HRS) was present in 19 patients, and 29 patients had refractory ascites (Table 1). Of note, the patients receiving antibiotics were either due to SBP prophylaxis or had already faded infection.

Table 2 summarises the biochemical and haemodynamic parameters of the patients before TIPS placement and 14 days after the placement. TIPS insertion slightly deteriorated liver function (as assessed by bilirubin, INR and ALT) but improved renal function in these patients (Table 2).

Patients were followed for at least 10 years. After 10 years, four of them were still alive, 38 patients had died, 5 patients had received a liver transplantation and 2 patients were lost from follow-up.

### Relationships between soluble TNFα receptors and portal hepatic pressure gradients before and after TIPS insertion

**Figure 1:**
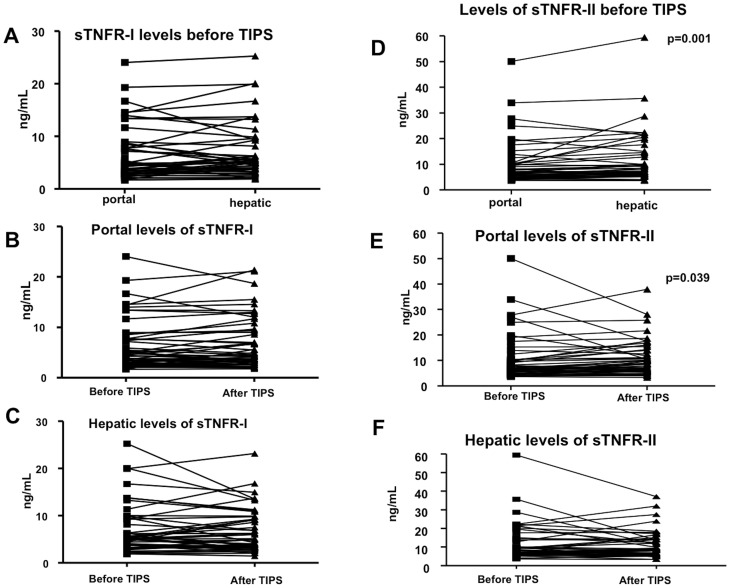
sTNFR levels in portal and hepatic venous blood before and after TIPS in all patients as well as stratified by Child classes. The levels of soluble TNFα receptors I (A–C) and II (D–E) in patients' portal and hepatic venous blood before and after TIPS. Mann-Whitney tests were used for comparison between the groups in the portal vein before TIPS. Data are shown paired.

Levels of soluble TNFα receptor I did not significantly differ between the portal vein and hepatic vein, neither before TIPS nor at the invasive check procedure 14 days after TIPS insertion (Figure 1A, B, C). Furthermore, portal decompression by TIPS did not induce any changes in soluble TNFR-I levels, neither in the portal vein nor in the hepatic vein. No association with the PHPG was observed before TIPS (data not shown). The levels of sTNFR-I in the hepatic veins correlated inversely with the PHPG after TIPS-placement (hepatic vein: r_s_ = −0.505, p = 0.012).

However, levels of soluble TNFα II receptor in the hepatic vein were significantly higher than in the portal vein (Figure 1D). Moreover, the levels of sTNFR-II in the portal vein were higher after TIPS insertion than before, but without any significant difference between the portal and hepatic veins (Figure 1E). This was accompanied by an inverse correlation of the levels of sTNFR-II in both compartments with PHPG (portal vein: r_s_ = −0.398, p = 0.040; hepatic vein: r_s_ = −0.447, p = 0.019).

### Correlations of soluble TNFα receptor levels with clinical and biochemical parameters

**Figure 2:**
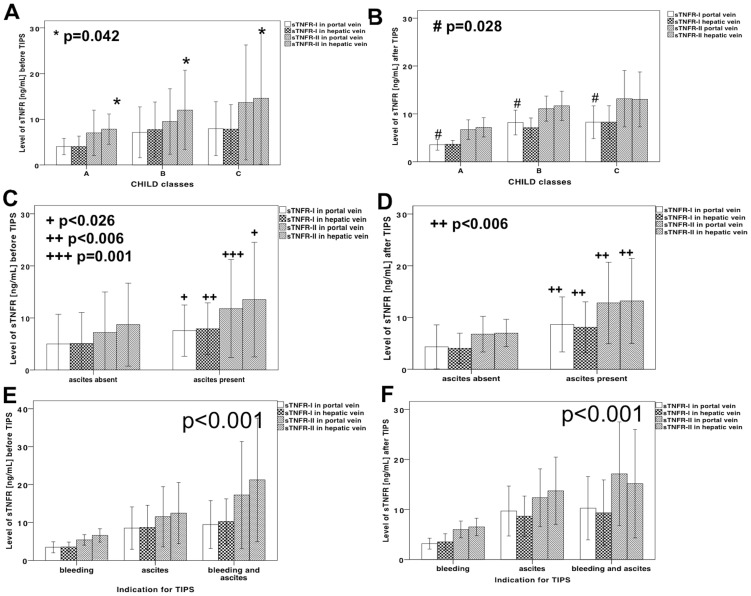
sTNFR levels in portal and hepatic venous blood before and after TIPS in patients stratified by presence of ascites, bleeding and indication for TIPS. Patients stratified according to their Child classes before TIPS in (A) and after TIPS in (B) showed high levels of soluble TNFα receptor II in the hepatic vein before TIPS, and soluble TNFα receptor I in the portal vein after TIPS. The levels of sTNFR before (C) and after (D) TIPS in patients with and without ascites. The levels of sTNFR before (E) and after (F) TIPS in patients with recurrent bleeding, refractory ascites or both indications. Mann-Whitney tests were used for comparison between the groups in the portal vein before TIPS, and Kruskal-Wallis tests were used for comparison between more than two groups. Data are shown as means +/− standard deviation.

Child score correlated with the levels of sTNFR-I in hepatic vein before TIPS (r_s_ = 0.364; p = 0.015) and in the portal vein after TIPS (r_s_ = 0.307; p = 0.036). The hepatic venous levels of sTNFR-II increased with increasing Child-classes before TIPS and the portal venous levels of sTNFR-I after TIPS insertion (Figure 2A, B).

The levels of both soluble TNFα receptors were significantly higher in patients with ascites (Figure 2C, D), and increased with the degree of ascites, while the levels of sTNFR were lower in the patients who received TIPS for recurrent variceal bleeding. Soluble TNFα receptors were significantly higher in patients with ascites than in patients receiving TIPS for recurrent variceal bleeding alone and were highest in patients with both bleeding and ascites (Figure 2E, F). These associations were similar before and after TIPS placement, which again suggests a pressure-independent relationship between sTNFR levels and complications and degree of liver dysfunction.

**Figure 3:**
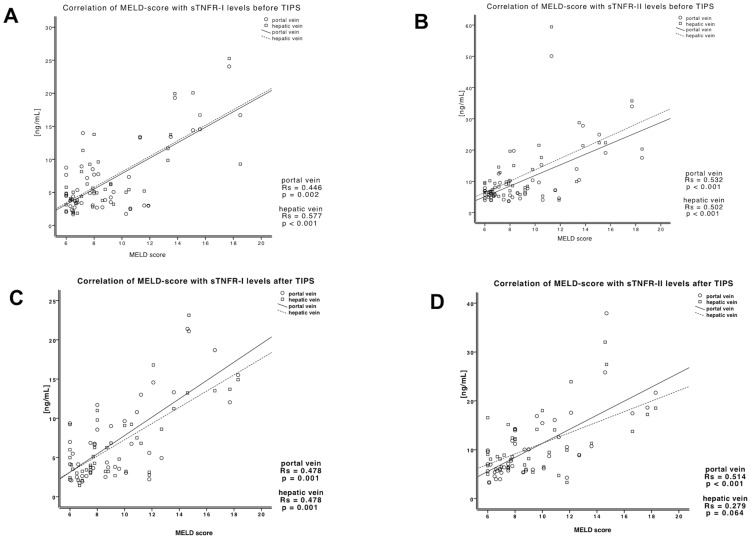
sTNFR levels in portal and hepatic venous blood before and after TIPS in patients correlated significantly with MELD score. The portal and hepatic venous levels of sTNFR-I before (A) and after (C) TIPS correlated with MELD score. The portal and hepatic venous levels of sTNFR-II before (B) and after (D) TIPS correlated with MELD score. Single data are presented including Spearman coefficient R_s_ and p-values.

The MELD score correlated significantly with the portal and hepatic venous levels of soluble TNFα receptor I and II before and after TIPS. This implies that levels of soluble TNFα receptors in cirrhotic patients with portal hypertension are dependent on liver function (Figure 3). The levels of serum creatinine were highly correlated with portal and hepatic venous levels of soluble TNFα receptors I and II before (sTNFR-I: portal vein: r_s_ = −0.657, p<0.001; hepatic vein: r_s_ = −0.772, p<0.001; sTNFR-II: portal vein: r_s_ = −0.687, p<0.001; hepatic vein: r_s_ = −0.674, p<0.001) and after TIPS (sTNFR-I: portal vein: r_s_ = −0.732, p<0.001; hepatic vein: r_s_ = −0.732, p<0.001; sTNFR-II: portal vein: r_s_ = −0.792, p<0.001; hepatic vein: r_s_ = −0.636, p = 0.001).

Markers of liver synthesis capacity, e.g. choline esterase (CHE) correlated negatively with both types of soluble TNFα receptors in the portal and hepatic veins both before (sTNFR-I: portal vein: r_s_ = −0.335, p = 0.026; hepatic vein: r_s_ = −0.582, p<0.001; sTNFR-II: portal vein: r_s_ = −0.427, p = 0.001; hepatic vein: r_s_ = −0.582, p<0.001) and after TIPS (sTNFR-I: portal vein: r_s_ = −0.575, p<0.001; hepatic vein: r_s_ = −0.575, p<0.001; sTNFR-II: portal vein: r_s_ = −0.601, p<0.001; hepatic vein: r_s_ = −0.489, p = 0.001). Interestingly, the correlation between albumin and the levels of soluble TNFR-I (portal vein: r_s_ = −0.473, p = 0.004; hepatic vein: r_s_ = −0.438, p = 0.009) and sTNFR-II (portal vein: r_s_ = −0.398, p = 0.013; hepatic vein: r_s_ = −0.341, p = 0.045) after TIPS was inverse.

Patients receiving antibiotics showed significantly higher levels of the sTNFR, while the use of beta-blocker, diuretics and lactulose had no effect on the levels of sTNFR (data not shown).

### The association of the soluble TNFα receptor levels with survival rates in patients receiving TIPS

**Figure 4:**
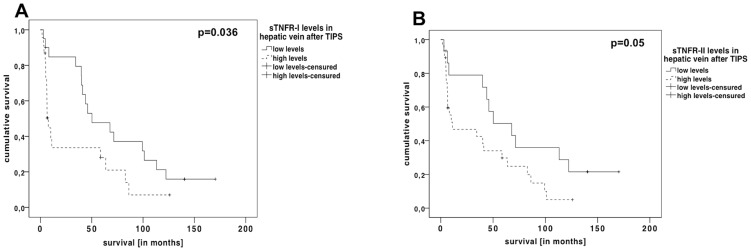
Kaplan-Meier curves of the patients stratified by low and high sTNFR levels. The survival of patients with levels of sTNFR-I higher than 4.5 ng/mL (A) and sTNFR-II above 7 ng/mL (B) after TIPS was significantly worse, than patients with circulating levels of these receptors below these threshholds.

Survival time correlated negatively with the levels of soluble TNFR-I in the portal vein before TIPS (r_s_ = −0.326, p = 0.027) and after TIPS, as well as in the hepatic vein after TIPS (portal vein: r_s_ = −0.329, p = 0.026; hepatic vein: r_s_ = −0.408, p = 0.007). The portal venous levels of soluble TNFR-II after TIPS correlated also negatively with survival time (r_s_ = −0.307, p = 0.036). When stratifying patients by lower or higher levels of soluble TNFRs in the hepatic vein (using the median to divide the groups), we observed significantly better survival rates in patients with low levels of the soluble TNFRs after TIPS insertion (Figure 4A, B).

In univariate analysis we analysed different parameters of disease severity and general characteristics (Table 3). Using a Cox regression analysis, including parameters (MELD, creatinine, CHE, Child-score) correlating significantly with the levels of soluble TNFRs the levels of soluble TNFR I in the hepatic vein after TIPS, together with the Child score, were independent predictors of survival (Table 4).

**Table 3:** Univariate time-to-event analysis of patients' characteristics (including variables of table 1 and 2).

Parameters	p-value
**sTNFR-I in hepatic vein after TIPS**	0.006
Child score	0.007
MELD score after TIPS	0.049
Creatinine after TIPS	0.028
CHE after TIPS	0.039

In the table are shown only significant variables.

MELD, Model for End Stage Liver Disease; CHE, choline esterase.

**Table 4:** Cox regression analysis (forward step-wise likelihood-quotient) using the significant variable from univariate analysis (table 3) to predict survival.

Variables	Significance	Hazard ratio	Confidence interval of hazard ratio
**Hepatic venous sTNFR-I after TIPS**	p = 0.004	1.120	1.036–1.211
Child score	p = 0.002	1.270	1.113–1.678

sTNFR-I levels in the hepatic vein after TIPS and Child score were the independent predictors for survival.

## Discussion

Our study showed that TIPS does not influence the TNFα system in patients with liver cirrhosis and portal hypertension. Furthermore, the hepatic and portal venous sTNFR levels determined before and early after TIPS placement are correlated to other parameters reflecting hepatic dysfunction, such as Child or MELD score. Furthermore, the levels of sTNFα receptors I in the hepatic vein after TIPS insertion were independent predictors of mortality.

Chronic liver disease, regardless of its aetiology, leads to complications, such as ascites and variceal bleeding from portal hypertension, which frequently require TIPS insertion. In these patients the portal pressure correlates with several inflammatory parameters indicative of an activated hepatic inflammatory status, which may contribute to portal hypertension [5]–[8]. In the present study the levels of sTNFR were higher in patients with ascites, suggesting that these receptors might reflect bacterial translocation and inflow of pathogen associated molecular patterns. Indeed, in cirrhotic patients the increased endotoxin influx into the portal vein leads to hepatic expression of soluble TNFα receptors even without evident clinical infection [31]. Different proinflammatory pathways (LPS/TLR/TNFα) activated by stimuli derived from the gut appear associated with the prognosis in addition to the classical prognostic parameters related to liver and kidney function [18]–[21], [23], [24], [26], [27], [36]. This can be seen in situations, where the prognosis deteriorates despite a tremendous drop in portal pressure by TIPS. Studies on the effects of a shunt procedure on inflammatory stimuli would be highly relevant both form a pathophysiological and prognostic point of view. The present study demonstrated a sustained activation of the TNF during a period of 14 days after TIPS placement. Obviously, TNFα-mediated inflammation and its negative influence on prognosis persist after TIPS.

Even though no major changes were observed in the levels of the sTNFRs levels after TIPS, the levels of sTNFRs correlated with parameters of liver dysfunction, confirming our previous data [31], [32]. In a previous study in patients with alcoholic liver cirrhosis receiving TIPS levels of endotoxin in the portal vein correlated with the levels of sTNFR in the hepatic vein, suggesting an association between endotoxin exposure and formation of sTNFR [31]. Interestingly, endotoxin levels in that study correlated with portal pressure and systemic hypotension. Of note, no correlation of sTNFR with portal pressure or portal-hepatic pressure gradient was observed before TIPS. These data again suggest that sTNFR levels are independent of the portal pressure, but rather reflect other factors contributing to deterioration of liver function. This assumption is supported by the fact that the levels of sTNFR-II in the portal vein increase shortly after TIPS insertion together with a slight increase of other markers for deterioration of liver function such as bilirubin, ALT, γ-GT and INR.

Thus sTNFR levels in the hepatic and portal vein may predict survival independent from portal pressure, even though difficult to sample portal or hepatic venous blood in a daily routine. However, sTNFRs in peripheral blood might be also suitable and their predictive potential should be evaluated in future studies in TIPS patients, as already shown by our group for sTNFR levels in the peripheral blood of non-TIPS patients [32]. Interestingly, hepatic sTNFR-I levels after TIPS are associated with long-term survival together with Child-score (Table 4). This study emphasises that survival of patients with severe portal hypertension is only partially reflected by their MELD score, also after TIPS placement. The TNFR levels add prognostic and important clinical information in these patients. Similarly to recently published data on acute-on-chronic liver failure, renal dysfunction and proinflammatory state seem to predict prognosis [37]. The levels of sTNFR correlate excellently with creatinine, and might reflect additional renal dysfunction in these patients. This study reflects the importance of inflammatory state in cirrhosis and indirectly it emphasizes the role of portal hypertension in the survival of patients with liver cirrhosis. This is supported by different studies showing an improved survival of patients receiving TIPS for refractory ascites [38] and as an emergency shunt for severe bleeding [39].

It is important to note that the included patients were without overt infection [4] since it is a prerequisite for TIPS placement. As a consequence, the levels of soluble TNFRs were much lower than in previous studies. This suggests that the sTNFRs are also infection-independent indicators of survival. In this situation sTNFRs might derive from the injured liver and cells driving hepatic inflammation and not from other foci, which might be a confounding factor in the above-mentioned studies [14], [21], [32]. In our study the levels of soluble TNFR-I showed the strongest prediction of survival in this group of patients, despite small number of patients.

In conclusion, the results of this study show that hepatic venous levels of soluble TNFR-I and II early after TIPS insertion correlate with mortality in patients with severe portal hypertension. The findings point to the role of chronic inflammation in patients with liver cirrhosis – independent from the degree of portal hypertension. The prognostic value of these markers may have potential in the management of patients with severe portal hypertension treated with TIPS. Prospective studies should further evaluate the impact of inflammation and the potential of sTNFRs for prognostic assessment of these patients.
